# Assessment of Women's Discretionary Salt Intake and Household Salt Utilization in Preparation for a Salt Fortification Trial in Oromia Region, Ethiopia

**DOI:** 10.1111/mcn.13768

**Published:** 2024-12-13

**Authors:** Isaac Agbemafle, Meseret Woldeyohannes, Masresha Tessema, Mengistu Fereja, Charles D. Arnold, Biniyam T. Banjaw, Alemayhu Hussen, Tadesse Kebebe, Yvonne E. Goh, Mandana Arabi, Homero Martinez, Christine M. McDonald, Kenneth H. Brown

**Affiliations:** ^1^ Department of Nutrition University of Rhode Island Kingston Rhode Island USA; ^2^ Department of Nutrition and Institute for Global Nutrition University of California Davis California USA; ^3^ Fred N. Binka School of Public Health University of Health and Allied Sciences Ho Volta Region Ghana; ^4^ Food Science and Nutrition Research Directorate Ethiopian Public Health Institute Gulelle Arbegnoch Street Addis Ababa Ethiopia; ^5^ Department of Pediatrics University of California, San Francisco Oakland California USA; ^6^ Global Technical Services Nutrition International Ottawa Ontario Canada

**Keywords:** discretionary salt, Ethiopia, folic acid, fortification, salt utilization, women

## Abstract

There is a high incidence of neural tube defects (NTDs) in Ethiopia and folate insufficiency, a primary risk factor for NTDs, is common among Ethiopian women of reproductive age (WRA). Folic acid fortification of salt has been proposed as a strategy to control these problems. In preparation for an intervention trial to assess the nutritional effects of folic acid‐fortified salt, we measured discretionary salt intakes among nonpregnant WRA using observed weighed food records, and we assessed household salt disappearance rates. We estimated the distribution of usual discretionary salt intake by adjusting for intra‐individual variability using the National Cancer Institute (NCI) method, and we simulated the potential effects of two levels of folic acid fortification (30 and 90 ppm folic acid) on folic acid intakes. Mean ± SD of usual discretionary salt intake was 6.8 ± 1.9 g/day. At the 95th percentile of usual discretionary salt intake, the higher fortification level would provide 918 µg folic acid/day, which is less than the tolerable upper intake level of 1000 µg/day. At the 5th percentile of usual discretionary salt intake, the lower fortification level would provide 124 µg folic acid/day, which should produce a statistically significant increase in red blood cell folate concentration. Estimated household salt utilization was 8.8 ± 6.1 g/person/day. These findings inform plans for a randomized, dose–response intervention trial of folic acid‐fortified salt and a possible future national program to mandate folic acid fortification of refined, edible salt in Ethiopia.

## Summary

- The mean usual discretionary salt intake by women in the study population was 6.8 g/day and the mean household salt utilization was 8.8 g/person/day.
- At the 95th percentile of usual discretionary salt intake, women consuming salt fortified with 90 µg folic acid/g salt would receive ~918 µg additional folic acid per day, which is less than the tolerable upper intake level of 1000 µg per day.
- At the 5th percentile of usual discretionary salt intake, women consuming salt fortified with 30 µg folic acid/g salt would receive ~124 µg additional folic acid per day, which should produce a statistically significant increase in red blood cell folate concentration.

## Graphical abstract

The mean usual discretionary salt intake was 6.8 g/day. At the 5th percentile of salt intake, women consuming salt with a lower fortification level (30 ppm) would receive ~124 µg/day folic acid. At the 95th percentile of salt intake, women consuming salt with a higher fortification level (90 ppm) would receive ~918 µg/day folic acid.

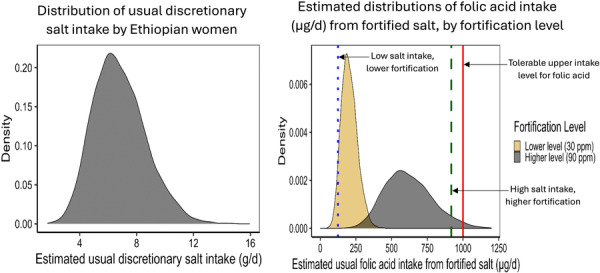

## 1 Introduction

Maternal folate insufficiency is the primary risk factor for neural tube defects (NTDs). In 2015, an estimated 260,100 pregnancies worldwide were affected by NTDs, including spina bifida, anencephaly, and related birth defects (prevalence = 18.6 per 10,000 live births) (Blencowe et al. 2018). In Ethiopia, the prevalence of NTDs is strikingly higher than the global average. Two recently published meta‐analyses of hospital‐based studies in Ethiopia found a mean birth prevalence of 63.3 per 10,000 (95% confidence interval [CI]: 50.9, 75.7) and 59.7 per 10,000 (95% CI: 42.1, 84.7) (Berhane Id and Belachew 2022; Bitew et al. 2020; Ssentongo et al. 2022); and one hospital‐based study in Addis Ababa, which also included pregnancy terminations, found an overall prevalence of 127.9 per 10,000 pregnancies after 12 weeks of gestation (Gedefaw, Teklu, and Tadesse 2018). Data from the 2023 Ethiopian Food and Nutrition Security survey showed that more than 74.6% of women have an elevated risk of NTDs, as indicated by folate insufficiency (RBC folate < 748 nmol/L) (Tessema et al. 2023).

Delivery of supplemental folic acid to women before they know they are pregnant is challenging, so food fortification with folic acid, a stable synthetic form of folate, is the primary NTD prevention strategy. Most national fortification programs add folic acid to cereal flours, and countries that have implemented folic acid fortification have reported substantial reductions in NTD prevalence (Centeno Tablante et al. 2019; Crider, Bailey, and Berry 2011). However, in many countries, poor rural populations have limited access to centrally processed, fortified cereal flours (Diosady, Mannar, and Krishnaswamy 2019). In Ethiopia, cereals are processed mostly in local community mills, which limits the potential reach of centrally processed fortified flour (Tessema et al. 2019). By contrast, almost all Ethiopians have regular access to iodized salt, which has resulted in significant declines in iodine deficiency (EPHI et al. 2021). Novel technologies now permit fortification of iodized salt with additional micronutrients, such as iron or folic acid, referred to as double‐fortified salt (DFS) or multiply fortified salt (MFS) (Modupe et al. 2021). Therefore, it may be possible to build on Ethiopia's successful salt iodization program to add folic acid to iodized salt. The Ethiopian Ministry of Health has recommended assessing the feasibility, acceptability, and nutritional effects of DFS containing iodine and folic acid (Tessema et al. 2019).

In preparation for a community‐based intervention trial to assess the effects of DFS containing iodine and folic acid versus iodized salt on the micronutrient status of nonpregnant women of reproductive age (WRA), we completed preliminary studies of women's discretionary salt intakes and household salt utilization in rural and semi‐urban communities of the Oromia region in Ethiopia. The purpose of these studies was to determine the appropriate level of folic acid fortification and the amount of salt to provide during the subsequent intervention trial. We decided a priori to set targets of 200 and 600 µg/day mean folic acid intakes for the two folic acid intervention arms of the planned dose–response trial, so as to bracket the currently recommended amount of additional folic acid intake (400 µg folic acid/day) for WRA (De‐Regil et al. 2015). We also planned to assess the amounts of additional folic acid that would be consumed by low‐ and high‐salt consumers in the respective study arms.

## 2 Methods

### 2.1 Study Design and Setting

This cross‐sectional study was conducted in the Gimbichu *woreda* (district) in the central highlands of the Oromia region of Ethiopia from June to August 2023 (planting season). The Gimbichu *woreda* was selected because of its relative proximity to Addis Ababa and year‐round accessibility by road (to facilitate frequent transfer of clinical specimens and food samples from the field site to the Ethiopian Public Health Institute [EPHI] laboratories), high contraceptive use, low potential for salt‐sharing among HHs, low use of folic acid‐fortified wheat flour, and acceptability of DFS, as determined during preliminary studies conducted by EPHI in the study area (Tesfaye, unpublished). The administrative centre of this *woreda* is Chefe Donsa, and Afaan Oromo is the primary local language. Gimbichu *woreda* is located 77 km from Addis Ababa, the capital of Ethiopia. The altitude of Chefe Donsa is ~2450 m, and the *woreda* has an estimated population of 86,902 people distributed across 35 *kebeles* (communities). According to *woreda*‐level data, about 27% of the population are WRA (15–49 years). The study was conducted in two semi‐urban and two rural *kebeles*.

### 2.2 Sampling and Sample Size

In collaboration with the Ministry of Health, we completed a census of all households (HHs) in the two semi‐urban *kebeles* surrounding the Chefe Donsa Health Centre. A list of HHs and WRA in the two rural *kebeles* was extracted from a digital list of HH members provided by the local health centre. HHs that did not have a WRA or did not plan to stay in the area for at least 1 month were excluded from the sampling frame. Potentially eligible WRA were listed sequentially by *kebele*, and an Excel random number generator was used to select individual women from each *kebele* in proportion to the population size. During the first home visit, field staff provided information on the study objectives and procedures, requested verbal consent for the screening interview, and administered the screening questionnaire to consenting women to assess their eligibility to participate in the study. We scheduled a second visit to the homes of potentially eligible women and requested their consent to participate in the full set of studies. We replaced women who were not eligible to participate or who withdrew from the study with another randomly selected WRA from the same *kebele* until we enroled the desired number of women.

All WRA aged 18–49 years were eligible to participate in the full study if they provided informed consent, as indicated by signature or thumb print, and they agreed to use the salt provided by the study team for the household salt disappearance study described below. We excluded women who were currently pregnant, and those reportedly suffering from an acute or chronic disease (such as diarrhoea, febrile illness or underlying metabolic disorder) that might affect their dietary intake or folate status or who were using medicines (like anticonvulsants and cancer treatments) that affect folate metabolism, as well as women who had medically prescribed restriction of salt intake. Using the combined lists of WRA and a population‐proportionate random sampling method, we enroled a sample size of 100 WRA. We determined the sample size based on logistical and cost considerations. Based on the average coefficient of variation of 44% observed in a similar community‐based study in India (Goh et al. 2023), we estimated that the proposed sample size would allow us to estimate the mean discretionary salt intake with a 95% CI and half width of ±0.6 g/day.

### 2.3 Data Collection

Field staff collected data on HH socio‐demographic characteristics, selected assets, and other socioeconomic indicators using an interviewer‐administered, semi‐structured questionnaire and assessed HH food insecurity using the HH Food Insecurity Access Scale (Coates, Swindale, and Bilinsky 2007). Dietitians carried out a full‐day dietary assessment, using weighed food records (WFRs), as described below; and repeated the WFRs in a subsample of 40 women approximately 1 week later to estimate the intra‐individual variation in intakes. Field staff also completed a 1‐week salt disappearance study in the homes of all women enroled in the study.

#### 2.3.1 Dietary Assessment

We carried out the WFRs as described previously (Goh et al. 2023). Briefly, on the dietary assessment day, we recorded the time and place of the observations and the amounts served and left over for each food and beverage item, including salt and water, to determine the amounts consumed. The dietary assessments were randomly distributed across weekdays and weekends. We weighed all ingredients, including salt and spice mixtures that were incorporated into mixed recipes, to the nearest 0.1 g, using dietary scales (Weiheng WH‐B23, China) that were calibrated daily with standard weights. We were interested in spice mixtures because they are common ingredients in Ethiopian recipes and an important source of discretionary salt. The dietitian stayed in the house of the WRA from approximately 6:00 a.m. to 8:00 p.m. and recorded the weights of all ingredients used in recipe preparation. We recorded the total pre‐ and post‐cooking weights of multi‐item recipes, and we calculated the amounts of each food item consumed as a proportion of the recipe weight. For any mixed dishes prepared the previous day, which occurred uncommonly, we estimated the recipe contents by participant recall of each ingredient. We also determined the composition of previously prepared spice mixtures and flour mixtures by recall. We weighed discrete food items (like pieces of fruit or biscuits) individually, including the rare occurrence of any salt added after serving. We measured salt added during food preparation and after serving food to determine the appropriate level of folic acid fortification of the DFS to be assessed in the subsequent intervention trial. The dietitians revisited the homes early the next morning to document any foods consumed after they left the homes the previous day; but night‐time food consumption was rarely reported. Finally, the dietitians solicited information about any maternal illnesses or changes in appetite on the study day; but none of the women reported such events. WFRs were completed using a paper‐based form, which was reviewed by the study supervisors and subsequently entered on electronic tablets using the Open Data Kit software app.

#### 2.3.2 Household Salt Disappearance

One to two weeks after completing the WFR, we assessed HH salt utilization by measuring the disappearance of a known amount of iodized, refined salt provided by the study team. The purpose of this study was to estimate the quantity of salt that we will need to deliver to each HH during the planned intervention trial. We delivered a 400 g package of salt to HHs with fewer than five members and a 700 g package to HHs with five or more members. We also supplied a plastic container for convenient storage of the salt provided by the study. We counselled the women to use only the study salt for all meals consumed by HH members, but not to use the salt for other purposes, such as animal feeding. A member of the study team called or visited the household mid‐week to check if any additional salt was needed. On the evening of the seventh day or early in the morning of the eighth day of the salt disappearance study, a member of the study team revisited the HH to weigh the remaining salt to a precision of 0.1 g and ask questions about the use of the study salt for HH meals and any other purposes during the previous week. We subtracted the final salt weight from the initial weight to determine the amount of HH salt utilization. During the HH salt disappearance study, we also collected information on the HH composition (age and sex of all HH members present during the week of the study) and the type of salt (i.e., coarse or refined) usually consumed by the HH, the different uses of salt, and the HH salt procurement and storage practices.

### 2.4 Data Analysis

We constructed a wealth index by using principal component analysis based on housing characteristics and ownership of selected assets (Vyas and Kumaranayake 2006); and we analysed household food insecurity access as described previously (Coates, Swindale, and Bilinsky 2007). We measured discretionary salt intake using the WFR data, specifically by summing the weights of all discretionary salt consumed from recipes or added at the time of food consumption. We used the SIMPLE SAS macro based on the National Cancer Institute (NCI) method to estimate the distribution of usual discretionary salt intake (Luo et al. 2021; NCI 2019). To demonstrate the potential effects of folic acid fortification of salt on inadequate and excessive intakes in the current sample, we used a two‐step model simulation based on the median usual discretionary salt intake (Luo et al. 2021; NCI 2019). The first step involved modelling the effect of a low dose of folic acid in the DFS to provide a mean of ~200 µg of additional folic acid per day, and the second step focused on the effect of a high dose in the DFS to provide a mean of ~600 µg of additional folic acid per day. The resulting concentrations were adjusted for the expected loss of folic acid over time based on the average annual temperature of 17°C in Chefe Donsa. Previous studies have shown that approximately 82% of the original folic acid in fortified salt remains after 6 months of storage at 25°C (Tesfaye, unpublished). Thus, the respective production targets for the DFS would be the initial amount of folic acid per gram of salt divided by 0.82.

We calculated HH salt utilization in relation to HH size and the adult female equivalent (AFE) factor. For these latter analyses, we used the FAO/WHO (2001) total energy expenditure (TEE) equation to estimate total HH energy requirements based on an assumed average adult female body weight of 55 kg, an assumed average adult male weight of 64 kg and a physical activity level factor of 1.5 for both females and males (FAO et al. 2001). For females and males below 18 years of age, we used the standard weights proposed by FAO/WHO to calculate their TEE (FAO et al. 2001). We calculated the WRA's energy requirement as a proportion of the total HH energy requirement, and we multiplied this proportion by the total daily HH salt utilization to express HH salt utilization in relation to the AFE factor (Weisell and Dop 2012). The total HH energy requirement was based on the HH composition data collected for each HH member present during the salt disappearance week.

We summarized socio‐demographic characteristics and salt procurement practices using frequency tables, and we described discretionary salt intake and HH salt utilization using the mean and SD. Discretionary salt intake and HH salt utilization were normally distributed, based on the Shapiro–Wilk test. We then applied linear mixed effect modelling with maximum likelihood estimation to assess the difference in apparent discretionary salt intakes by demographic covariates (e.g., fixed effects: age, semi‐urban/rural residence, weekend data collections). The independent variables were occupation, religion, marital status, socioeconomic status, household food insecurity status, household size, and vitamin and mineral supplement use. We included a random effect of participant to account for the dependence between observations on the same woman. We completed the data analyses using Stata version 16.1 (StataCorp, College Station, Texas) with significance set at *p* < 0.05.

### 2.5 Ethical Statement

The study protocol was approved by the Institutional Review Boards of the Ethiopian Public Health Institute and the University of California, Davis. The study was registered at clinicaltrials.gov (NCT1834465‐1).

## 3 Results

**Figure 1:**
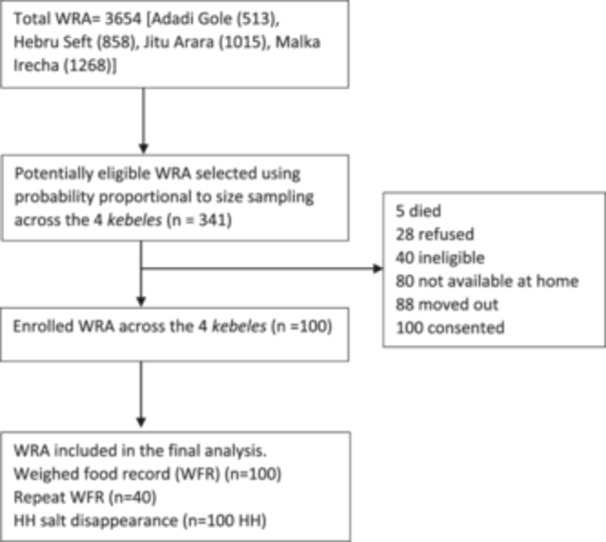
Flow of study participants.

Figure 1 illustrates the flow of participants through the study. On average, the women were 31 years of age and approximately one‐fourth of them had received no formal education (Table 1). The average household size was 5, and approximately one‐third of the HHs were moderately or severely food insecure.

**Table 1:** Background characteristics of women of reproductive age in study communities, Oromia region, Ethiopia (*N* = 100).

Variable	Mean or frequency
Age (years) [mean ± SD]	30.5 ± 8.3
20–29	53
30–39	31
40–49	16
Educational level	
No formal education	26
Any primary	39
Primary completed	16
Secondary/technical/vocational or higher	19
Occupation	
Housewife	56
Trader	16
Farmer	11
Professional	8
Others (i.e., artisans, skilled labourer)	9
Religion	
Orthodox Christian	79
Protestant Christian	15
Muslim/traditional	7
Marital status	
Married/living together	75
Divorced/separated	11
Widowed	2
Never married/single	12
Household size [mean ± SD]	4.9 ± 1.8
2–4	43
5–7	49
8–10	8
Housing wall	
Natural walls (thatch, mud, cane, palm)	84
Finished walls (brick, concrete, cement)	15
Corrugated metal walls	1
Housing floor	
Earth/sand/dung	61
Cement	35
Palm/bamboo	2
Vinyl/asphalt strips	2
Housing roof	
Roofing shingles/corrugated metal	68
Thatch/palm leaf/palm/sack/cardboard	13
Wood	9
Plastic roof	5
Cement	4
Ceramic tiles	1
Source of light	
Electricity	91
Solar lantern	6
Kerosene lamp	2
Rechargeable flashlight, torch or lantern	1
Household food insecurity	
None	56
Mild	9
Moderate	26
Severe	9
Used vitamin and mineral supplements in the past 1 month	
No	100
Fortifiable flour use^a^	
No	77
Yes	1
Don't know	22

^a^ Suitable for being fortified (industrially produced).

The main types of salt available in households were coarse salt (82%), refined salt (9%), or both coarse and refined salt (9%, Table 2). Nearly half (47%) of the women did not know whether the main salt available in the home was fortified with iodine. Regardless of the type of salt used, about two‐thirds of the women reportedly used salt just for food preparation, while about one‐fourth used it for both food preparation and for animal feeds (Table 2). Salt is mostly sourced from the market in its original packaging and stored in the home in a separate container with lid (76%). About one in five women reported sharing salt occasionally, mostly with neighbours in the same compound, at most once a month.

**Table 2:** Reported salt procurement practices among women of reproductive age in study communities in Oromia region, Ethiopia.

Variables	Frequency *n* (%)
Main types of household salt	
Coarse salt only	82 (82.0)
Refined salt only	9 (9.0)
Both coarse and refined salt	9 (9.0)
Main salt iodized	
No	20 (20.0)
Yes	33 (33.0)
Don't know	47 (47.0)
Coarse salt uses (*n* = 85)	
Food preparation	57 (67.1)
Food preparation and season food at table	4 (4.7)
Food preparation and animal feed preparation	24 (28.2)
Refined salt uses (*n* = 15)	
Food preparation	9 (60.0)
Food preparation and animal feed preparation	6 (40.0)
Source of iodized salt (*n* = 33)	
Market	21 (63.6)
Shop/kiosk	12 (36.4)
Salt labelled as iodized	
Yes, in original package	20 (20.0)
Yes, in plastic bag	13 (13.0)
Don't know/not sure	67 (67.0)
Checks salt iodization logo	
No	78 (78.0)
Yes	9 (9.0)
Don't know	13 (13.0)
Salt storage	
Container with lid	76 (76.0)
Container without lid	10 (10.0)
Same bag/sachet purchased in	9 (9.0)
Plastic bag	5 (5.0)
Salt sharing	
No	82 (82.0)
Yes, same compound	15 (15.0)
Yes, nearby structure	3 (3.0)
Frequency (days/month) of salt sharing (*n* = 18)	1 ± 1

**Figure 2:**
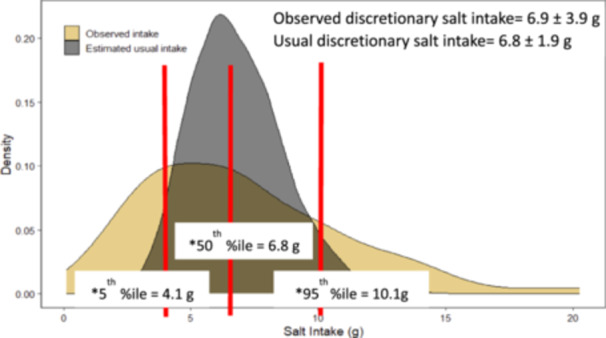
Distributions of observed discretionary salt intake and estimated usual discretionary salt intake after adjustment for intra‐individual variation in intake (g/person/day) among women of reproductive age in Oromia region, Ethiopia. *5th, 50th and 95th percentiles are based on the usual discretionary salt intake (grey coloured or conical shaped curve).

The women's mean ± SD discretionary salt intake observed during the WFRs was 6.9 ± 3.9 g/day (Figure 2). After adjusting for intra‐individual variation in discretionary salt intake, the mean (SD) usual discretionary salt intake was 6.8 ± 1.9 g/day. Of the total discretionary salt intake, 6.2 ± 3.6 g was from salt added during food preparation, and 0.7 ± 0.9 g was from salt included in the spice mixtures used during cooking. Salt was rarely added to foods at the time of consumption.

Based on the observed mean usual intake of discretionary salt, salt should be fortified with folic acid at concentrations of ~30 µg folic acid per g of salt (30 ppm) and ~90 µg folic acid per gram of salt (90 ppm) to achieve the desired mean intakes of ~200 µg/day or ~600 µg/day in the respective lower and higher folic acid arms in the planned intervention trial (Table 3). We expected ~18% folic acid losses over time; thus, the respective production targets for the DFS are 37 µg folic acid per gram of salt and 110 µg folic acid per gram of salt (Table 3), which should result in mean folic acid intakes at the desired levels over the course of the study. Considering the observed variability in usual discretionary salt intakes (Figure 2, as indicated by the grey or conical shaped curve), women at the 95th percentile of usual salt intake who receive the higher level of folic acid fortification would consume 918 µg additional folic acid per day. Women in the lower folic acid fortification arm with the usual discretionary salt intake at the 5th percentile would consume 124 µg additional folic acid per day.

**Table 3:** Proposed levels of folic acid (FA) fortification (µg/g salt) for Phase 2 by targeted amount of folic acid intake, and estimated range of resulting folic acid intakes (µg/day)^a^.

	Iodine only	Iodine + 200 µg folic acid	Iodine + 600 µg folic acid
Desired mean folic acid concentration^a^ (µg/g)	0	30	90
Target initial folic acid concentration^b^ (µg/g)	0	37	110
Estimated 5th percentile folic acid intake^a^ (µg/day)	0	124	373
Estimated 95th percentile folic acid intake^a^ (µg/day)	0	306	918

^a^ Based on median usual discretionary salt intake of 6.8 g/day, we determined the levels of fortification needed to deliver previously set targets of 200 and 600 µg/day of additional folic acid intakes for the two folic acid arms of the future trial (200 µg/day of folic acid ÷ 6.8 g/day of salt = 30 µg of folic acid per gram of salt; 600 µg/day of folic acid ÷ 6.8 g/day of salt = 90 µg of folic acid per gram of salt). We then multiplied these concentrations by 5th and 95th percentiles of usual salt intake to estimate the amount of additional folic acid that would be consumed.

^b^ Target folic acid concentration is based on the folic acid concentration divided by 0.82 to compensate for the assumed 18% losses over time under field conditions.

Three‐quarters of the women reported that their study salt intakes during the HH salt utilization studies were the same as usual, although some (21%) indicated that their intake of the study salt was lower than usual, mainly because they felt that less of the refined salt provided for the HH utilization studies was needed to produce the same taste as coarse salt. The mean daily total HH salt utilization was 38.8 ± 22.0 g/day and this increased in relation to household size (Table 4). There was not a substantial difference in HH salt utilization when expressed per person (8.8 ± 6.1 g/day) or per AFE (8.4 ± 6.0 g/day). On average, the mean salt utilization per person was about 2 g greater than the women's observed discretionary salt intake of 6.9 g/day. There were no significant associations between discretionary salt intake or HH salt utilization and socio‐demographic characteristics (Supporting Information S1: Table S1).

**Table 4:** Household salt utilization among women of reproductive age in study communities in Oromia region, Ethiopia.

Salt utilization	Mean ± SD
Household salt utilization (g/day/HH)	38.8 ± 22.0
2–3‐member household (*n* = 21)	30.5 ± 18.3
4–5‐member household (*n* = 46)	37.8 ± 17.7
6–7‐member household (*n* = 25)	44.8 ± 30.7
8+ member household (*n* = 8)	47.4 ± 13.9
Salt utilization (g/day/per person)^a^	8.8 ± 6.1
Salt utilization (g/day/per AFE)^b^	8.4 ± 6.0
Uses of study salt	*N* = 100
Food preparation^c^	85
Food preparation and seasoning food^c^	15
Reported study salt intake during salt disappearance study	
Same as usual salt intake^c^	75
Lower than usual salt intake^c^	21
Higher than usual salt intake^c^	4

^a^ Per capita salt utilization was expressed as the difference in the weight of study salt on Day 1 and Day 7 or 8 of the salt disappearance study divided by the number of study days and the total number of HH members.

^b^ AFE—Adult female equivalent was calculated as the woman's energy requirement in proportion to the total HH energy requirement multiplied by the total daily HH salt utilization.

^c^ Values are expressed as a percentage of the total sample size (*N* = 100).

## 4 Discussion

We assessed women's discretionary salt intakes, using full‐day WFRs and found that the women's mean usual discretionary salt intake was 6.8 g/day. At the 95th percentile of usual discretionary salt intake, women who receive the higher level of folic acid fortification would consume 918 µg additional folic acid per day, which is less than the tolerable upper intake level (UL) of 1000 µg per day (Turck et al. 2023). According to the North American dietary reference intake publication, the UL for folate for adults is 1000 µg/day of folate from fortified food or supplements (IOM, 1998). In other words, the UL only considers additional folic acid from fortified foods and supplements, not dietary folate. The reason for this is because of uncertainties in food folate contents and the assumption that folate intake from intrinsic food folate is low relative to the UL. At the 5th percentile of usual discretionary salt intake, women in the lower folic acid fortification arm would consume 124 µg additional folic acid per day. Thus, almost all women in the upcoming intervention trial would consume enough of the fortified salts to produce a detectable increase in their RBC folate concentration (Hao et al. 2008), and very few would consume more than the UL. Thus, by using a range of folic acid intakes in the planned dose–response intervention trial, we will be able to estimate the appropriate level of folic acid fortification to achieve the desired red blood cell folate concentration indicative of folate sufficiency (≥ 748 nmol/L) for most women in the study population.

The mean usual discretionary salt intake of the study participants is generally consistent with the reported mean salt intake (estimated from single spot urine samples) of 7.4 g/day among adult females who participated in the Ethiopian 2015 STEP survey (Challa et al. 2017). This observation is consistent with previous studies that concluded that salt intakes worldwide have not changed substantially in recent years despite recommendations to curtail salt intake to decrease the burden of noncommunicable diseases (Santos et al. 2021).

We also measured HH salt utilization rates by weighing HH salt disappearance over a period of 1 week and expressed the results both as per capita salt utilization and in relation to AFE. Per capita salt utilization was used to account for the number of persons eating the meals prepared using the study salt. The AFE was used to estimate WRA's salt intake in relation to the composition of the HH, considering the age, sex, and estimated body size and physical activity level of each HH member. The per capita salt utilization and AFE were greater than the observed discretionary salt intakes by WRA, likely because the salt was used for other purposes, such as the preparation of spice mixtures for future use. These findings are consistent with a previous study of household salt disappearance in Cambodia (Chan et al. 2021), which found that salt disappearance overestimated observed salt intake, although the main explanation for this difference in Cambodia was the use of salt for fish preservation. Sharing of salt between HHs occurred rarely (less than once per month in just 20% of HHs), so it should not affect the interpretation of the planned intervention trial.

Based on the pattern of HH salt utilization, we determined that we could meet the needs of approximately 85% of the households (mean + 1 SD of HH salt utilization) during the upcoming intervention trial by delivering 1 kg of salt every 2 weeks. We plan to package the salt in 500 g canisters, so that we can deliver an additional canister in cases where individual HHs require more than 1 kg of salt over the 2‐week period. In future intervention trials, it will be important to counsel participants on restricting use of the study salt only to HH members so the trial results can be interpreted in relation to actual intakes.

Notably, most HHs reported using coarse salt rather than refined salt. Currently, coarse salt is used more commonly because of its greater availability and lower cost (Tesfaye, unpublished). These issues could limit acceptance and uptake of DFS unless the refined salt is partially subsidized, or fortification is mandated for both coarse and refined salt. Current national guidelines on salt iodization recommend fortifying all food‐grade salt for human and animal consumption, including salt for food processing (Yusufali et al. 2022), so the same guidelines presumably could be applied for DFS. This issue of salt choices should not pose a problem for the forthcoming intervention trial, as almost all HHs were willing to use refined salt that was provided gratis. However, in the future it will be important to develop messaging to explain and promote the use of DFS and to modify the current national salt iodization logo to include folic acid to ensure consumer acceptance and demand for DFS.

Our estimates of women's discretionary salt intake, from both salt consumption and HH salt utilization methods, are lower than the estimated global per capita salt consumption estimate of 9–12 g/day, but consistent with estimates of 6.7 g/day reported for Africa (WHO 2021, 2023). This is not surprising, as the global salt consumption estimates are based on national food balance sheets, which account for salt production, imports, and exports, but may not fully capture how much of the salt is used for food preservation, animal feeds, and industrial applications or is wasted (FAO 2018). Both current estimates of salt consumption are greater than the WHO recommendation of < 5 g per capita per day (PAHO and WHO 2021). Nevertheless, if countries are ultimately successful in reducing discretionary salt intakes, it will be possible to increase the concentration of fortificants to maintain the targeted intakes of added micronutrients. Therefore, salt remains a good candidate for delivering micronutrients, while respecting efforts to reduce salt consumption, as salt is universally consumed in fairly consistent amounts within populations. Moreover, our study and others (Allen et al. 2006; Diosady, Mannar, and Krishnaswamy 2019) found that salt intakes do not vary by socioeconomic status or other individual characteristics.

This study had several strengths. In particular, we selected a representative sample of the proposed study population to be included in the future intervention trial, so that we could determine the suitability of the study site, the appropriate levels of folic acid fortification for the trial, and the amount of salt to deliver to study participants and their HHs. Second, we used rigorous observational methods to measure women's discretionary salt intake and HH salt utilization. Third, we used the NCI method to adjust for intra‐individual (day‐to‐day) variation in discretionary salt intake (Luo et al. 2021; NCI 2019), and thereby estimate the distribution of women's usual intakes. Finally, we collected additional information on salt utilization practices and preferences and descriptive information on the women and their HHs to assess possible factors associated with salt consumption and utilization. One potential weakness of the study is the sample size, which was restricted by available funding and time for completion. Another limitation is that we could not assess salt intake of other HH members, urban dwellers and from other regions of Ethiopia. If results of the phase 2 trial are favourable, salt consumption by other HH members, urban dwellers and from other regions of Ethiopia as well as folic acid intake from fortifiable foods would need to be considered before establishing the dose of folic acid to be mandated by the national DFS program in Ethiopia. Although the final sample size was sufficient to allow for adjustment of intra‐individual variability in discretionary salt intakes, our failure to detect any factors associated with salt intakes may have been due to a limited sample size. Nevertheless, any potential associations that were undetected would be of small magnitude and would not affect ultimate plans for the intervention. Additionally, these associations could be re‐evaluated with the larger sample size that will be recruited for the intervention trial.

## 5 Conclusion

Evidence suggests that mandatory food fortification is a cost‐effective and efficacious intervention to improve population micronutrient status (Allen et al. 2006), and salt has been proposed as a potential vehicle for folic acid fortification in Ethiopia (Woldeyohannes et al. 2023). NTDs are prevalent in Ethiopia and a major cause of infant and young child mortality and disability (Tessema et al. 2019), and folic acid fortification is the primary prevention strategy used worldwide. Folic acid fortification of salt is an attractive intervention option in Ethiopia because of its feasibility (Modupe et al. 2021) and consumer acceptability (Tesfaye, unpublished). Salt is locally processed in a reasonably small number of factories, and folic acid fortification can build on the positive experience with the existing national salt iodization program in Ethiopia (Yusufali et al. 2022). The upcoming community‐based intervention trial will assess the longer‐term acceptability, safety, and nutritional impact of the DFS containing iodine and folic acid.

## Author Contributions

Isaac Agbemafle drafted and revised the manuscript. Meseret Woldeyohannes, Masresha Tessema, Biniyam T. Banjaw, Christine M. McDonald and Kenneth H. Brown conceived the study and wrote the initial study protocol. Homero Martinez and Mandana Arabi commented on the study design and implementation. Isaac Agbemafle, Mengistu Fereja, Tadesse Kebebe, Yvonne E. Goh and Alemayhu Hussen facilitated implementation of the study. Meseret Woldeyohannes, Masresha Tessema and Kenneth H. Brown were involved in study coordination. Isaac Agbemafle developed the statistical analysis plan, and Isaac Agbemafle and Charles D. Arnold performed the statistical analyses. All authors read and approved the final manuscript and agreed to be accountable for all aspects of the work.

## Conflicts of Interest

Homero Martinez and Mandana Arabi receive salaries from Nutrition International, Canada. Kenneth H. Brown is a part‐time consultant to the Bill & Melinda Gates Foundation. All other authors declare no conflict of interest.

## Supporting information

Supporting information.

## Data Availability

Data are available upon request from the study team. The complete data set and study forms will be made available online at osf.io within 3 years after the completion of data collection.
