# Supplementary material for: Assessment of Women's Discretionary Salt Intake and Household Salt Utilization in Preparation for a Salt Fortification Trial in Oromia Region, Ethiopia
Source: Matern Child Nutr. 2024 Dec 13;21(2):e13768. doi: 10.1111/mcn.13768 (PMC11956044; doi:10.1111/mcn.13768)
Supplement: Supplementary file 1 — Supporting information. [file MCN-21-e13768-s001.docx]

**SUPPLEMENTARY TABLE 1:** Predictors of discretionary salt intake among women of reproductive age in Oromia region, Ethiopia

| Variable | B (95% CI) |
| --- | --- |
| Age (years) [Mean ± SD] | -0.02 (-0.10, 0.07) |
| Educational level |  |
| No education | Ref |
| Any primary | -0.88 (-2.74, 0.99) |
| Primary completed | 0.14 (-2.36, 2.63) |
| Secondary or higher | -0.28 (-2.85, 2.29) |
| Occupation |  |
| Housewife | Ref |
| Farmer | 0.59 (-1.64, 2.82) |
| Trader | 1.35 (-0.59, 3.28) |
| Others (i.e. artisans, skilled laborer, professionals) | 1.55 (-0.54, 3.64) |
| Marital status |  |
| Single/ widowed | Ref |
| Married/ Living together | 1.32 (-0.39, 3.03) |
| Wealth index |  |
| First quintile | Ref |
| Second quintile | 1.83 (-0.36, 4.03) |
| Third quintile | 0.55 (-1.60, 2.71) |
| Forth quintile | 1.03 (-116, 3.22) |
| Fifth quintile | 0.47 (-1.85, 2.80) |
| Household food insecurity |  |
| None | Ref |
| Mild | -0.99 (-3.32, 1.34) |
| Moderate | -1.26 (-3.01, 0.48) |
| Severe | -1.41 (-3.88, 1.05) |
| Household size | -0.20 (-0.74,0.35) |
| Vitamin and mineral supplement use |  |
| No | Ref |
| Yes | -0.42 (-1.85, 1.01) |
